# 2-(Trimethyl­siloxy)adamantane-2-carbonitrile

**DOI:** 10.1107/S1600536808043559

**Published:** 2008-12-24

**Authors:** Richard Betz, Peter Klüfers, Peter Mayer

**Affiliations:** a Ludwig-Maximilians Universität, Department Chemie und Biochemie, Butenandtstrasse 5–13 (Haus D), 81377 München, Germany

## Abstract

In the crystal structure of the title compound, C_14_H_23_NOSi, cyclic dimeric units are established by two very weak hydrogen bonds of the type C—H⋯N with an H⋯N distance which is only slightly shorter than the sum of the van der Waals radii of 2.75 Å. The graph-set descriptor on the unitary level is *R*_2_^2^(14) for the cyclic dimer.

## Related literature

For a general synthesis of trimethyl­silan­yloxy-substituted cyano­hydrines, see Evans *et al.* (1974 ▶). For the crystal structure of a related compound, see Hickmott *et al.* (1985 ▶). For hydrogen-bond motifs, see: Bernstein *et al.* (1995 ▶); Etter *et al.* (1990 ▶).
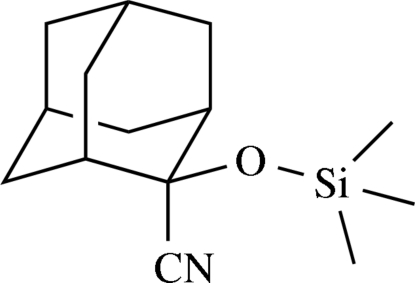


## Experimental

### 

#### Crystal data

C_14_H_23_NOSi
*M* *_r_* = 249.42
Triclinic, 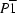

*a* = 6.712 (2) Å
*b* = 9.440 (3) Å
*c* = 12.439 (2) Å
α = 106.19 (2)°
β = 102.35 (2)°
γ = 100.34 (3)°
*V* = 715.0 (4) Å^3^
*Z* = 2
Mo *K*α radiation
μ = 0.15 mm^−1^
*T* = 200 (2) K
0.38 × 0.34 × 0.18 mm

#### Data collection

Oxford Xcalibur diffractometer
Absorption correction: multi-scan (*CrysAlis RED*; Oxford Diffraction, 2005 ▶) *T*_min_ = 0.91, *T*_max_ = 0.97
5687 measured reflections
2872 independent reflections
2097 reflections with *I* > 2σ(*I*)
*R*_int_ = 0.019

#### Refinement

*R*[*F* ^2^ > 2σ(*F* ^2^)] = 0.036
*wR*(*F* ^2^) = 0.099
*S* = 1.06
2872 reflections
157 parameters
H-atom parameters constrained
Δρ_max_ = 0.23 e Å^−3^
Δρ_min_ = −0.18 e Å^−3^

### 

Data collection: *CrysAlis CCD* (Oxford Diffraction, 2005 ▶); cell refinement: *CrysAlis RED* (Oxford Diffraction, 2005 ▶); data reduction: *CrysAlis RED*; program(s) used to solve structure: *SHELXS97* (Sheldrick, 2008 ▶); program(s) used to refine structure: *SHELXL97* (Sheldrick, 2008 ▶); molecular graphics: *ORTEPIII* (Burnett & Johnson, 1996 ▶) and *Mercury* (Macrae *et al.*, 2006 ▶); software used to prepare material for publication: *SHELXL97* and *PLATON* (Spek, 2003 ▶).

## Supplementary Material

Crystal structure: contains datablocks global, I. DOI: 10.1107/S1600536808043559/lh2736sup1.cif

Structure factors: contains datablocks I. DOI: 10.1107/S1600536808043559/lh2736Isup2.hkl

Additional supplementary materials:  crystallographic information; 3D view; checkCIF report

## Figures and Tables

**Table 1:** Hydrogen-bond geometry (Å, °)

*D*—H⋯*A*	*D*—H	H⋯*A*	*D*⋯*A*	*D*—H⋯*A*
C10—H10⋯N^i^	1.00	2.68	3.516 (3)	141

Symmetry code: (i) 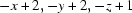.

**Fig. 1.**
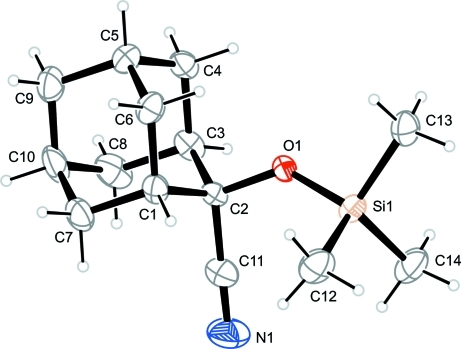
The molecular structure of the title compound, with atom labels and anisotropic displacement ellipsoids (drawn at 50% probability level) for non-H atoms.

**Fig. 2.**
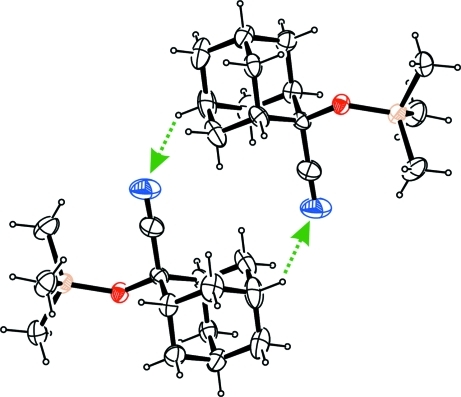
Intermolecular interactions in the crystal structure of the title compound, viewed approximately along [-1 0 0].

**Fig. 3.**
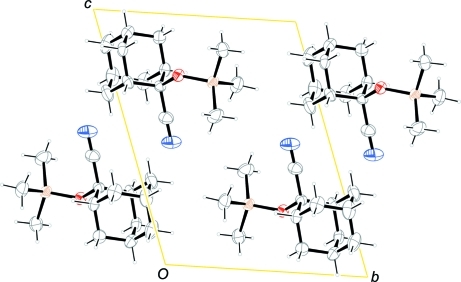
The packing of the title compound, viewed along [-1 0 0].

## supplementary crystallographic information

### Comment

2-Trimethylsilanyloxy-adamantane-2-carbonitrile was prepared as an intermediate in the synthesis of (2-adamantyl)-glycolic acid.

In the crystal packing of the title compound, C_14_H_23_NOSi, dimeric units are established by two very weak hydrogen bonds of the type C–H···N with an H···N distance of 2.68 Å, which is only slightly shorter than the sum of the van-der-Waals radii of 2.75 Å (see Fig. 2).

The graph-set descriptor on the unitary level for the cyclic dimer is *R*^2^_2_(14) (Etter *et al.*, 1990; Bernstein *et al.*, 1995).

The packing of the title compound is shown in Figure 3.

In the molecule the cyano group and the trimethylsilanyloxy group reside on the same C atom resembling a similar compound apparent in the literature (Hickmott *et al.*, 1985). The methyl groups on the silicon atom adopt a nearly staggered conformation with respect to the substituents on the functionalized carbon atom. Bond lengths and angles in the carbocycle are in good agreement with the ones observed for other adamantane-derived compounds.

### Experimental

The title compound was prepared in adoption of a published procedure (Evans *et al.*, 1974) upon Lewis-acid catalyzed addition of trimethylsilylcyanide to 2-adamantanone.

Crystals suitable for X-ray analysis were obtained directly from the crystallized reaction product obtained after distillation under reduced pressure.

### Refinement

Carbon-bound H atoms were placed in calculated positions (C—H 1.00 Å for bridgehead C atoms, C—H 0.99 Å for methylene groups and C—H 0.98 Å for methyl groups) and were included in the refinement in the riding model approximation, with *U*(H) set to 1.2*U*_eq_(C) for bridgehead C atoms and methylene groups and 1.5*U*_eq_(C) for methyl groups.

### Crystal data

**Table:**

C_14_H_23_NOSi	*Z* = 2
*M**_r_* = 249.42	*F*(000) = 272
Triclinic, *P*1	*D*_x_ = 1.159 Mg m^−^^3^
Hall symbol: -P 1	Mo *K*α radiation, λ = 0.71073 Å
*a* = 6.712 (2) Å	Cell parameters from 3060 reflections
*b* = 9.440 (3) Å	θ = 4.1–26.3°
*c* = 12.439 (2) Å	µ = 0.15 mm^−^^1^
α = 106.19 (2)°	*T* = 200 K
β = 102.35 (2)°	Block, colourless
γ = 100.34 (3)°	0.38 × 0.34 × 0.18 mm
*V* = 715.0 (4) Å^3^	

### Data collection

**Table:**

Oxford Xcalibur diffractometer	2872 independent reflections
Radiation source: fine-focus sealed tube	2097 reflections with *I* > 2σ(*I*)
graphite	*R*_int_ = 0.019
ω scans	θ_max_ = 26.3°, θ_min_ = 4.1°
Absorption correction: multi-scan (*CrysAlis RED*; Oxford Diffraction, 2005)	*h* = −8→8
*T*_min_ = 0.91, *T*_max_ = 0.97	*k* = −11→8
5687 measured reflections	*l* = −15→15

### Refinement

**Table:**

Refinement on *F*^2^	Primary atom site location: structure-invariant direct methods
Least-squares matrix: full	Secondary atom site location: difference Fourier map
*R*[*F*^2^ > 2σ(*F*^2^)] = 0.036	Hydrogen site location: inferred from neighbouring sites
*wR*(*F*^2^) = 0.099	H-atom parameters constrained
*S* = 1.06	*w* = 1/[σ^2^(*F*_o_^2^) + (0.0562*P*)^2^] where *P* = (*F*_o_^2^ + 2*F*_c_^2^)/3
2872 reflections	(Δ/σ)_max_ < 0.001
157 parameters	Δρ_max_ = 0.23 e Å^−^^3^
0 restraints	Δρ_min_ = −0.18 e Å^−^^3^

### Special details

**Table:**

Experimental. CrysAlis RED, Oxford Diffraction Ltd., Version 1.171.32.5 (release 08-05-2007 CrysAlis171 .NET) (compiled May 8 2007,13:10:02) Empirical absorption correction using spherical harmonics, implemented in SCALE3 ABSPACK scaling algorithm.

### Fractional atomic coordinates and isotropic or equivalent isotropic displacement parameters (Å^2^)

**Table:**

	*x*	*y*	*z*	*U*_iso_*/*U*_eq_	
Si	0.23305 (7)	0.50326 (5)	0.26051 (3)	0.03296 (15)	
O	0.32961 (14)	0.66707 (11)	0.24268 (8)	0.0301 (3)	
N	0.6710 (3)	0.7967 (2)	0.51049 (13)	0.0652 (5)	
C1	0.6875 (2)	0.72644 (17)	0.22407 (13)	0.0330 (4)	
H1	0.7090	0.6266	0.2304	0.040*	
C2	0.5289 (2)	0.77526 (16)	0.29081 (12)	0.0284 (3)	
C3	0.5002 (2)	0.93025 (16)	0.28158 (13)	0.0345 (4)	
H3	0.3995	0.9644	0.3256	0.041*	
C4	0.4126 (2)	0.91123 (17)	0.15282 (13)	0.0363 (4)	
H41	0.2755	0.8337	0.1198	0.044*	
H42	0.3888	1.0090	0.1454	0.044*	
C5	0.5668 (3)	0.86187 (18)	0.08534 (14)	0.0407 (4)	
H5	0.5084	0.8500	0.0014	0.049*	
C6	0.5977 (3)	0.70911 (17)	0.09590 (13)	0.0380 (4)	
H61	0.4608	0.6313	0.0629	0.046*	
H62	0.6956	0.6746	0.0512	0.046*	
C7	0.8992 (2)	0.8475 (2)	0.27499 (16)	0.0479 (4)	
H71	0.9567	0.8602	0.3583	0.057*	
H72	1.0016	0.8142	0.2332	0.057*	
C8	0.7134 (3)	1.04922 (18)	0.33150 (16)	0.0516 (5)	
H81	0.7719	1.0612	0.4146	0.062*	
H82	0.6941	1.1490	0.3267	0.062*	
C9	0.7790 (3)	0.9803 (2)	0.13533 (18)	0.0560 (5)	
H91	0.8784	0.9473	0.0912	0.067*	
H92	0.7608	1.0792	0.1279	0.067*	
C10	0.8675 (3)	0.9988 (2)	0.26269 (17)	0.0536 (5)	
H10	1.0058	1.0774	0.2954	0.064*	
C11	0.6099 (2)	0.78913 (19)	0.41541 (14)	0.0416 (4)	
C12	0.4270 (3)	0.38626 (19)	0.26501 (15)	0.0512 (5)	
H121	0.4627	0.3606	0.1908	0.077*	
H122	0.3660	0.2922	0.2781	0.077*	
H123	0.5549	0.4441	0.3286	0.077*	
C13	0.0044 (3)	0.40872 (19)	0.13013 (14)	0.0471 (4)	
H131	−0.0928	0.4746	0.1273	0.071*	
H132	−0.0687	0.3114	0.1341	0.071*	
H133	0.0533	0.3901	0.0599	0.071*	
C14	0.1455 (3)	0.5381 (2)	0.39525 (15)	0.0545 (5)	
H141	0.2677	0.5922	0.4633	0.082*	
H142	0.0794	0.4404	0.4014	0.082*	
H143	0.0434	0.6002	0.3923	0.082*	

### Atomic displacement parameters (Å^2^)

**Table:**

	*U*^11^	*U*^22^	*U*^33^	*U*^12^	*U*^13^	*U*^23^
Si	0.0365 (3)	0.0333 (3)	0.0339 (3)	0.00931 (19)	0.01383 (19)	0.01512 (19)
O	0.0236 (5)	0.0318 (6)	0.0344 (6)	0.0042 (4)	0.0052 (4)	0.0141 (5)
N	0.0664 (11)	0.0837 (12)	0.0349 (9)	0.0152 (9)	−0.0005 (8)	0.0168 (8)
C1	0.0293 (8)	0.0309 (8)	0.0452 (9)	0.0136 (7)	0.0133 (7)	0.0164 (7)
C2	0.0249 (7)	0.0287 (8)	0.0268 (7)	0.0055 (6)	0.0017 (6)	0.0066 (6)
C3	0.0319 (8)	0.0254 (8)	0.0398 (9)	0.0106 (7)	0.0049 (7)	0.0027 (7)
C4	0.0312 (8)	0.0272 (8)	0.0483 (10)	0.0073 (7)	0.0021 (7)	0.0161 (7)
C5	0.0407 (9)	0.0429 (10)	0.0436 (9)	0.0084 (8)	0.0121 (8)	0.0235 (8)
C6	0.0413 (9)	0.0375 (9)	0.0408 (9)	0.0128 (7)	0.0209 (7)	0.0127 (7)
C7	0.0267 (8)	0.0544 (11)	0.0670 (12)	0.0122 (8)	0.0097 (8)	0.0280 (9)
C8	0.0480 (11)	0.0279 (9)	0.0594 (12)	0.0033 (8)	−0.0066 (9)	0.0050 (8)
C9	0.0388 (10)	0.0535 (12)	0.0840 (14)	0.0039 (9)	0.0149 (10)	0.0419 (11)
C10	0.0253 (8)	0.0424 (10)	0.0826 (14)	−0.0048 (7)	−0.0007 (9)	0.0251 (10)
C11	0.0385 (9)	0.0457 (10)	0.0362 (10)	0.0126 (8)	0.0051 (8)	0.0095 (8)
C12	0.0609 (12)	0.0443 (10)	0.0617 (12)	0.0231 (9)	0.0235 (10)	0.0273 (9)
C13	0.0434 (10)	0.0402 (10)	0.0525 (11)	−0.0013 (8)	0.0119 (8)	0.0158 (8)
C14	0.0629 (12)	0.0661 (12)	0.0489 (11)	0.0169 (10)	0.0317 (10)	0.0285 (9)

### Geometric parameters (Å, °)

**Table:**

Si—O	1.6594 (11)	C6—H61	0.9900
Si—C13	1.8491 (19)	C6—H62	0.9900
Si—C12	1.8540 (17)	C7—C10	1.526 (2)
Si—C14	1.8562 (16)	C7—H71	0.9900
O—C2	1.4200 (17)	C7—H72	0.9900
N—C11	1.143 (2)	C8—C10	1.535 (2)
C1—C6	1.530 (2)	C8—H81	0.9900
C1—C7	1.535 (2)	C8—H82	0.9900
C1—C2	1.5411 (19)	C9—C10	1.516 (3)
C1—H1	1.0000	C9—H91	0.9900
C2—C11	1.489 (2)	C9—H92	0.9900
C2—C3	1.5415 (19)	C10—H10	1.0000
C3—C4	1.531 (2)	C12—H121	0.9800
C3—C8	1.532 (2)	C12—H122	0.9800
C3—H3	1.0000	C12—H123	0.9800
C4—C5	1.523 (2)	C13—H131	0.9800
C4—H41	0.9900	C13—H132	0.9800
C4—H42	0.9900	C13—H133	0.9800
C5—C9	1.525 (2)	C14—H141	0.9800
C5—C6	1.530 (2)	C14—H142	0.9800
C5—H5	1.0000	C14—H143	0.9800
			
O—Si—C13	102.98 (7)	C10—C7—C1	109.55 (13)
O—Si—C12	111.91 (7)	C10—C7—H71	109.8
C13—Si—C12	110.75 (8)	C1—C7—H71	109.8
O—Si—C14	110.55 (7)	C10—C7—H72	109.8
C13—Si—C14	110.49 (9)	C1—C7—H72	109.8
C12—Si—C14	109.99 (8)	H71—C7—H72	108.2
C2—O—Si	133.06 (9)	C3—C8—C10	109.94 (13)
C6—C1—C7	109.53 (13)	C3—C8—H81	109.7
C6—C1—C2	108.61 (11)	C10—C8—H81	109.7
C7—C1—C2	110.13 (13)	C3—C8—H82	109.7
C6—C1—H1	109.5	C10—C8—H82	109.7
C7—C1—H1	109.5	H81—C8—H82	108.2
C2—C1—H1	109.5	C10—C9—C5	109.63 (15)
O—C2—C11	108.81 (12)	C10—C9—H91	109.7
O—C2—C1	111.04 (11)	C5—C9—H91	109.7
C11—C2—C1	109.74 (12)	C10—C9—H92	109.7
O—C2—C3	108.61 (11)	C5—C9—H92	109.7
C11—C2—C3	109.88 (12)	H91—C9—H92	108.2
C1—C2—C3	108.74 (12)	C9—C10—C7	110.15 (16)
C4—C3—C8	109.24 (14)	C9—C10—C8	110.01 (14)
C4—C3—C2	108.53 (12)	C7—C10—C8	108.35 (15)
C8—C3—C2	109.88 (12)	C9—C10—H10	109.4
C4—C3—H3	109.7	C7—C10—H10	109.4
C8—C3—H3	109.7	C8—C10—H10	109.4
C2—C3—H3	109.7	N—C11—C2	178.65 (18)
C5—C4—C3	110.11 (12)	Si—C12—H121	109.5
C5—C4—H41	109.6	Si—C12—H122	109.5
C3—C4—H41	109.6	H121—C12—H122	109.5
C5—C4—H42	109.6	Si—C12—H123	109.5
C3—C4—H42	109.6	H121—C12—H123	109.5
H41—C4—H42	108.2	H122—C12—H123	109.5
C4—C5—C9	110.22 (14)	Si—C13—H131	109.5
C4—C5—C6	108.75 (12)	Si—C13—H132	109.5
C9—C5—C6	109.20 (14)	H131—C13—H132	109.5
C4—C5—H5	109.6	Si—C13—H133	109.5
C9—C5—H5	109.6	H131—C13—H133	109.5
C6—C5—H5	109.6	H132—C13—H133	109.5
C1—C6—C5	109.87 (12)	Si—C14—H141	109.5
C1—C6—H61	109.7	Si—C14—H142	109.5
C5—C6—H61	109.7	H141—C14—H142	109.5
C1—C6—H62	109.7	Si—C14—H143	109.5
C5—C6—H62	109.7	H141—C14—H143	109.5
H61—C6—H62	108.2	H142—C14—H143	109.5
			
C13—Si—O—C2	−160.82 (12)	C3—C4—C5—C6	60.43 (16)
C12—Si—O—C2	−41.84 (13)	C7—C1—C6—C5	−59.17 (16)
C14—Si—O—C2	81.13 (13)	C2—C1—C6—C5	61.14 (16)
Si—O—C2—C11	−37.90 (16)	C4—C5—C6—C1	−60.41 (17)
Si—O—C2—C1	83.00 (14)	C9—C5—C6—C1	59.90 (18)
Si—O—C2—C3	−157.48 (10)	C6—C1—C7—C10	58.48 (17)
C6—C1—C2—O	58.21 (15)	C2—C1—C7—C10	−60.90 (18)
C7—C1—C2—O	178.15 (11)	C4—C3—C8—C10	−58.61 (17)
C6—C1—C2—C11	178.56 (12)	C2—C3—C8—C10	60.35 (18)
C7—C1—C2—C11	−61.50 (17)	C4—C5—C9—C10	59.23 (18)
C6—C1—C2—C3	−61.23 (15)	C6—C5—C9—C10	−60.17 (18)
C7—C1—C2—C3	58.71 (15)	C5—C9—C10—C7	60.26 (19)
O—C2—C3—C4	−59.91 (15)	C5—C9—C10—C8	−59.12 (19)
C11—C2—C3—C4	−178.82 (12)	C1—C7—C10—C9	−59.28 (19)
C1—C2—C3—C4	61.05 (15)	C1—C7—C10—C8	61.10 (18)
O—C2—C3—C8	−179.29 (12)	C3—C8—C10—C9	59.34 (19)
C11—C2—C3—C8	61.79 (17)	C3—C8—C10—C7	−61.12 (18)
C1—C2—C3—C8	−58.34 (16)	O—C2—C11—N	44 (7)
C8—C3—C4—C5	58.71 (16)	C1—C2—C11—N	−77 (7)
C2—C3—C4—C5	−61.09 (16)	C3—C2—C11—N	163 (7)
C3—C4—C5—C9	−59.25 (17)		

### Hydrogen-bond geometry (Å, °)

**Table:**

*D*—H···*A*	*D*—H	H···*A*	*D*···*A*	*D*—H···*A*
C10—H10···N^i^	1.00	2.68	3.516 (3)	141

Symmetry codes: (i) −*x*+2, −*y*+2, −*z*+1.

## References

[bb1] Bernstein, J., Davis, R. E., Shimoni, L. & Chang, N.-L. (1995). *Angew. Chem. Int. Ed. Engl.* **34**, 1555–1573.

[bb2] Burnett, M. N. & Johnson, C. K. (1996). *ORTEPIII* Report ORNL-6895. Oak Ridge National Laboratory, Tennessee, USA.

[bb3] Etter, M. C., MacDonald, J. C. & Bernstein, J. (1990). *Acta Cryst.* B**46**, 256–262. 10.1107/s0108768189012929 2344397

[bb4] Evans, D. A., Carroll, G. L. & Truesdale, L. K. (1974). *J. Org. Chem.* **39**, 914–917.

[bb5] Hickmott, P. W., Wood, S. & Murray-Rust, P. (1985). *J. Chem. Soc. Perkin Trans. 1*, pp. 2033–2038.

[bb6] Macrae, C. F., Edgington, P. R., McCabe, P., Pidcock, E., Shields, G. P., Taylor, R., Towler, M. & van de Streek, J. (2006). *J. Appl. Cryst.* **39**, 453–457.

[bb7] Oxford Diffraction (2005). *CrysAlis CCD* and *CrysAlis RED* Oxford Diffraction Ltd, Abingdon, United Kingdom.

[bb8] Sheldrick, G. M. (2008). *Acta Cryst.* A**64**, 112–122. 10.1107/S0108767307043930 18156677

[bb9] Spek, A. L. (2003). *J. Appl. Cryst.* **36**, 7–13.

